# Reactive Oxygen Species, Antioxidant Responses and Implications from a Microbial Modulation Perspective

**DOI:** 10.3390/biology11020155

**Published:** 2022-01-18

**Authors:** Peiman Zandi, Ewald Schnug

**Affiliations:** 1 International Faculty of Applied Technology, Yibin University, Yibin 644000, China; peiman.zandi@yibinu.edu.cn; 2 Department of Life Sciences, Institute for Plant Biology, Technical University of Braunschweig, 38106 Braunschweig, Germany

**Keywords:** reactive oxygen species, environmental stress, oxidative damage, antioxidants

## Abstract

**Simple Summary:**

Environmental conditions are subject to unprecedented changes due to recent progressive anthropogenic activities on our planet. Plants, as the frontline of food security, are susceptible to these changes, resulting in the generation of unavoidable byproducts of metabolism (ROS), which eventually affect their productivity. The response of plants to these unfavorable conditions is highly intricate and depends on several factors, among them are the species/genotype tolerance level, intensity, and duration of stress factors. Defensive mechanisms in plant systems, by nature, are concerned primarily with generating enzymatic and non-enzymatic antioxidants. In addition to this, plant-microbe interactions have been found to improve immune systems in plants suffering from drought and salinity stress.

**Abstract:**

Plants are exposed to various environmental stresses in their lifespan that threaten their survival. Reactive oxygen species (ROS), the byproducts of aerobic metabolism, are essential signalling molecules in regulating multiple plant developmental processes as well as in reinforcing plant tolerance to biotic and abiotic stimuli. However, intensified environmental challenges such as salinity, drought, UV irradiation, and heavy metals usually interfere with natural ROS metabolism and homeostasis, thus aggravating ROS generation excessively and ultimately resulting in oxidative stress. Cellular damage is confined to the degradation of biomolecular structures, including carbohydrates, proteins, lipids, pigments, and DNA. The nature of the double-edged function of ROS as a secondary messenger or harmful oxidant has been attributed to the degree of existing balance between cellular ROS production and ROS removal machinery. The activities of enzyme-based antioxidants, catalase (CAT, EC 1.11.1.6), monodehydroascorbate reductase (MDHAR, E.C.1.6.5.4), dehydroascorbate reductase (DHAR, EC 1.8.5.1), superoxide dismutase (SOD, EC 1.15.1.1), ascorbate peroxidase (APX, EC 1.11.1.11), glutathione reductase (GR, EC 1.6.4.2), and guaiacol peroxidase (GPX, EC 1.11.1.7); and non-enzyme based antioxidant molecules, ascorbate (AA), glutathione (GSH), carotenoids, α-tocopherol, prolines, flavonoids, and phenolics, are indeed parts of the defensive strategies developed by plants to scavenge excess ROS and to maintain cellular redox homeostasis during oxidative stress. This review briefly summarises current knowledge on enzymatic and non-enzymatic antioxidant machinery in plants. Moreover, additional information about the beneficial impact of the microbiome on countering abiotic/biotic stresses in association with roots and plant tissues has also been provided.

## 1. Introduction

Constant changes in environmental conditions exacerbates unfavorable, stressful conditions such as salinity, drought, extreme temperatures, waterlogging, or heavy metal stress, severely affecting plant growth, development, and yield through inducing changes in plant physiological and biochemical characteristics [1,2].

Plenty of studies have shown that plants are prone to generate both highly reactive oxygen free radicals and slightly reactive non-radicals of oxygen derivatives after subjection to various environmental biotic and/or abiotic stresses [3,4,5]. This generation, which corresponds to nearly 1–2% of the total plant’s oxygen (O_2_) consumption [6], is highly premised on the presence and functioning of respiratory burst oxidase homologues (RBOHs); and the plant NADPH oxidases [7,8,9]. The oxidative products are collectively referred to as the reactive oxygen species (ROS) and predominantly include superoxide anion radical (O_2_^•−^), hydroxyl radical (OH^•^), perhydroxyl radical (HO_2_^•^), singlet oxygen (^1^O_2_) and hydrogen peroxide (H_2_O_2_) [3,10]. The interconversion rate among unwanted ROS byproducts is extremely high, making them functionally variable and potent oxidants considering their disparity in stability, reactivity, and ability to transport through/across biological membranes [11]. Both ROS types (radical/non-radical) are naturally formed at basal levels in the course of many aerobic metabolic processes such as chloroplast and mitochondrial electron transport chains [12,13], during photorespiration in peroxisomes [14], or in apoplastic spaces [15,16]. 

Generally, ROS basic levels generated under optimal environmental conditions cannot cause cellular damage due to the expression of stress-responsive genes [17]. This level of ROS generation, based on some evidence, has been suggested to be connected to their limited natural involvement in developmental processes [18] or the regulation of morphogenetic processes associated with phytohormones such as cytokinins and auxins [19]. On the other hand, stressful environmental conditions excessively accelerate cellular ROS concentrations [20] at levels exceeding the antioxidant scavenging capacities [11,17] employed by plants to neutralise excess ROS production [21]. This feature potentially leads to oxidative stress along with damage to membrane lipids, proteins, and nucleic acids and eventually results in cell death [4,22,23]. Abiotic stress-induced programmed cell death (PCD) is a genetically controlled process in which the intensity of detrimental factors is harnessed through increased ROS generation [24]. Environmental stress factors involved in exerting disturbance on the delicate balance between ROS production and ROS removal pathways include but not limited to drought, salinity, high irradiance, extreme temperatures, heavy metals, pollution, and pathogen infection [22,25,26]. 

Plant adaptation to the oxidative stress caused by elevated ROS concentrations is often consistent with many key factors, among which are stress duration and severity, instantaneous cellular energy status, plant growth stages, ROS cellular level, and antioxidant capacity [27]. Generally, ROS-scavenging systems in plants are comprised of ascorbate (AA), glutathione (GSH), carotenoids, α-tocopherol, prolines, flavonoids, and phenolic compounds as non-enzymatic antioxidants, monodehydroascorbate reductase (MDHAR), dehydroascorbate reductase (DHAR), superoxide dismutase (SOD), catalase (CAT), ascorbate peroxidase (APX), glutathione reductase (GR), and guaiacol peroxidase (GPX) as well as low molecular mass (LMS) antioxidants and/or enzymatic antioxidants [20,21,27,28,29]. It is well reported that increased activity of antioxidant enzymes or non-enzymatic antioxidants in response to unprecedented environmental stresses helps to ameliorate the degree of damage caused by oxidative stress [5,26]. For instance, in green bean (*Phaseolus vulgaris* L.) plants affected by salinity stress, a subtle increase in the activity of CAT and APX antioxidants was observed in salt-tolerant GS57 and salt-sensitive 4F-89 genotypes, which was consistent with increased fresh and dry weight in these plants [30]. Similarly, an overall increase was reported in antioxidant enzymes (i.e., APX, SOD, GPX, CAT and GR) of some traditional rice plants suffering from drought stress [31]. The upregulation of AA, GSH, phenolics, phytochelatins (as GSH oligomers), and sugars as non-enzyme-based antioxidants was induced in Antarctica in *Colobanthus quitensis* (Kunth) Bartl by copper stress treatment [32]. 

Plant growth-promoting rhizobacteria (PGPRs) are known to be beneficial microorganisms and play a contributing role in reinforcing plant response to stressful conditions [33]. Typically, after colonising the rhizosphere or endo-rhizosphere of plants, the PGPRs adopt several direct and/or indirect mechanisms to promote plant growth at the expense of tackling abiotic stresses [33,34,35,36]. Some of these mechanisms include the induction of osmolyte accumulation [37], the activation of the antioxidant defence system [38], the up/downregulation of stress-responsive genes [39,40], and alteration in root morphology in acquisition of stress tolerance [41,42]. PGPR-induced drought or salinity tolerance in biological systems mainly occurs though induction of physical and chemical alterations in plants, which are collectively referred to as PGPR-induced systemic tolerance (IST) [43,44]. For instance, a consortium of three PGPR strains, viz. *Bacillus subtilis* SM21, *Bacillus cereus* AR156, and *Serratia* sp. XY21, have been shown to enhance the drought tolerance of cucumber (*Cucumis sativus* L.) plants by increasing the activity of SOD and mitigating the expression of genes encoding the cytosolic APX in cucumber leaf tissues [45].

This review aimed to further extend our understanding of antioxidants and their function in protecting plants against oxidative stress, as well as of plant–microbe interactions that confer abiotic stress tolerance in planta. Therefore, we provided a relatively comprehensive overview of major enzymatic and non-enzymatic antioxidants, pointed out the importance of PGPRs in alleviating plant stress, and summarised some current knowledge on them. 

## 2. Enzymatic Defensive Mechanisms

Preventive mechanisms against ROS overaccumulation are the antioxidant capacity by which enzymatic activities confer plant tolerance to adverse environmental conditions [11,46]. Many genetic studies emphasised the significance of expressing these antioxidant enzymes in increasing plant survival rate [47,48]. There are several main ROS-eliminating enzymatic systems in the site of production (subcellular compartments) in plants, namely, SOD, CAT, APX, GPX, GR, MDHAR, and DHAR [11,26].

### 2.1. Superoxide Dismutase

This omnipresent metalloenzyme, which can be found in almost all aerobic organisms including plants, in all intracellular organelles and apoplastic spaces [49], is actively present at the forefront of defence against oxidative damage from ROS [4,50]. The primary function of superoxide dismutase (SOD, EC 1.15.1.1) is to dismutate superoxide radicals (O^•−^_2_) into H_2_O_2_ and O_2_, such that it eventually wards off the formation of OH^•^ by the metal-catalysed Haber–Weiss reaction [26,49,51]. In other words, the catalysing activity of this enzyme regulates the amount of O_2_ and H_2_O_2_, which are the Haber–Weiss reaction substrates, and reduces the risk of producing highly active OH^−^ radicals. Based on the metal ion it binds, three main isozymes/isoforms (SODs) of this enzyme have been introduced thus far in the *Arabidopsis thaliana* genome: Cu/Zn-SOD gene localised to the chloroplast thylakoids (CSD_2_), peroxisomes (CSD_3_), and the cytosol (CSD_1_), Fe-SOD gene localised to the chloroplast stroma (FSD_1_), cytosol (FSD_1_), thylakoids (FSD_2_, FSD_3_), and the nucleus (FSD_1_), and Mn-SOD (MSD_1_) gene localised to the mitochondrial matrix [11,46,49,52,53,54]. These isoforms have been identified as the main ROS scavengers under drought stress conditions [55]. Additionally, nutrient deficiency (K, P, Mg, Ca, S or N) increased SOD functioning and introduced new isoforms in maize plants [56]. The existence of coherent coordination between peroxidase (an H_2_O_2_ scavenger) and enhancement of the activity of chloroplastidial Fe-SOD usually leads to plant protection when CO_2_ assimilation is intensely reduced; or otherwise results in elevated cytotoxicity in the Haber–Weiss reaction [57,58]. Such coordination during exposure to oxidative stress has already been observed in leaves of tea [59], tobacco [60], onion [61] and cotton [62] plants. In addition to the patented purification and characterisation techniques (e.g., anion exchange chromatography and ammonium sulfate precipitation) [63], another possible approach to increasing SOD levels in leaves of stress-tolerant plant species would be exposing them to environmental stresses such as low temperature, water deficit, or salinity [64,65].

Regardless of SOD types, they are initially encrypted in the nucleus and then transferred to various organs [46,66]. In chloroplast, the SOD enzyme is attached/bonded to the thylakoid membrane as a solution in the stroma [49]. The former one dismantles the superoxide radical immediately at the site of production, and the stroma-soluble form converts the superoxide radicals released in the stroma into H_2_O_2_ [4]. Most of the research conducted on SOD was centred on how environmental stresses or genotypes might affect the enzymatic antioxidants alone [57,67] or combined [68,69,70] with other plant items. In a study by Boguszewska et al. [71], it was found that abiotic stress conditions can upregulate the formation of SODs in potato plants. Heavy metals such as zinc oxide nanoparticles (ZnO-NP) escalate NADP-oxidase activity and thus increase the production of superoxide radicals [58]. Apart from their beneficial antioxidative function in plants suffering from salt [72], oxidative [52], or photooxidative [73] stresses, there are indications showing that SODs have an effect on root development [53], germination [46], flowering [74], and not least, chloroplast development [52]. 

### 2.2. Catalase

As a Fe-containing homotetrameric enzyme, catalase (CAT, EC 1.11.1.6) is responsible for the catalysation of H_2_O_2_ overproduced during light respiration or photorespiration in peroxisomes, H_2_O_2_ produced during β-oxidation of fatty acids in glyoxysomes, or H_2_O_2_ produced by SOD [4,75]. In all these cases, CAT mediates the dismutation of oxygenated water (H_2_O_2_) into water and oxygen in an energy-efficient way [26]. Peroxisomes are the core sites of CAT activity in response to H_2_O_2_ production as a result of oxidative stress, purine catabolism, photorespiration, and not least the β-oxidation of fatty acids [10,76]. Nevertheless, a more recent report highlights their presence in the cytosol, chloroplast, mitochondria, and other subcellular compartments [77]. Despite having a high specificity for H_2_O_2_, CAT showed a low affinity for organic peroxides [77]. One of the unique characteristics of this enzyme is its high turnover rate and low demand for reducing equivalents [26]. This enzyme is encoded by different CAT genes in several plant species [11]. For example, these include seven CAT genes reported in cotton (*Gossypium hirsutum* L.) [78], four in cucumber (*Cucumis sativus* L.) [79], and in rice (*Oryza sativa L*.) [80], three in *(Arabidopsis thaliana* L.) Heynh. [81], pumpkin (*Cucurbita Linn*.) [82], maize (*Zea mays* L.) [83], Tex-Mex tobacco (*Nicotiana plumbaginifolia* Viviani) [84], two in common barley (*Hordeum vulgare* L.) [85], and in tomato (*Lycopersicon esculentum* Mill.) [86], and one in sweet potato (*Ipomoea batatas* L.) Poir. [87], and in castor bean (*Ricinus communis* L. cv. Hale) [88], among others (Table 1). 

In Arabidopsis, the first two (CAT_1_ and CAT_2_) genes are predominantly present in glyoxysomes, peroxisomes and cytosol, and the latter (CAT_3_) is present in mitochondria and cytosol, with no presence reported in chloroplasts [4,77]. Recent reports indicate that these genes are primarily expressed in seeds (CAT_1_, CAT_2_, CAT_3_), pollens (CAT_1_), roots (CAT_2_), photosynthetic tissues (CAT_2_), and vascular tissues (CAT_3_) [26,121,122,123,124]. The involvement of CATs in the growth, development, or senescence of organs such as roots [122], shoots, flower parts (ovule, pollen), seeds [121,123], and leaves [77,125] has been explicitly documented.

Because of low affinity of CATs to H_2_O_2_, only high concentrations of H_2_O_2_ have the possibility of being eliminated by CATs [126]. In particular, CAT_1_ is responsible for scavenging H_2_O_2_ under abiotic stress conditions, and CAT_2_ and CAT_3_ for removing H_2_O_2_ to maintain ROS homeostasis both in light and darkness [78]. Stimulation of enzymatic antioxidants such as CATs has been reported in maize plants exposed to arsenate and arsenite [127]. Leaf senescence significantly impacts the concentration reduction of CAT_2_ rather than CAT_1_ in tobacco [110]. As for the model plant, *Arabidopsis*, all three CATs are engaged with photooxidation responses [11,124,128], CAT_1_ is involved in the plant’s response to drought and salt stress [129], CAT_2_ is implicated in toxic metal [130], heat [131], cold and salt [132] stress responses and CAT_3_ is incorporated in drought stress responses [133].

### 2.3. Ascorbate Peroxidase

The ascorbate peroxidase (APX, EC 1.1.11.1) enzyme functions in the ascorbate-glutathione cycle and regularly exists in chloroplasts (stroma and thylakoid), mitochondria, peroxisomes, cytosols, vacuoles, and the apoplast [4,11]. Five main isoforms of the APX family in rice plants are named after their constituent amino acids and their subcellular localisation [134]. Recent studies on the *Arabidopsis* genome revealed the presence of nearly nine putative APX genes, localised in the cytosol (APX_1_, APX_2_, APX6), peroxisomes (APX_3_ and APX_5_), chloroplast stroma (sAPX), chloroplast thylakoid (tAPX) [135,136], and mitochondria (sAPX) [137], with two of them (APC_4_ and APX_7_) being annotated as genes lacking H_2_O_2_ detoxifying activity and pseudogene, respectively [138]. The critical importance of cytosolic APXs is not so much linked to their conference of plant tolerance to cold [139], salinity (APX_2_), high light (APX_2_), heat and drought (APX_1_) [140,141] stresses as to their depletion (e.g., APX_1_) which tends to inactivate chloroplastic H_2_O_2_ detoxification [142,143].

**Figure 1:**
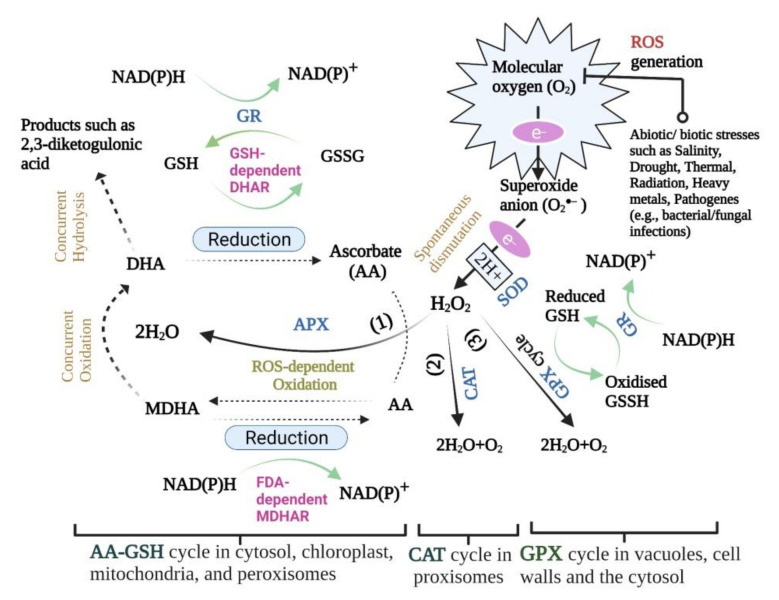
Schematic model showing the reactive oxygen species (ROS) generation in plants as well as the ascorbate-glutathione (ascorbic acid/AA-GSH) and the guaiacol peroxidase (GPX) cycles. AA is oxidised by ROS and converted into monodehydroascorbate (MDHA). A set of three enzymes, including FAD-dependent monodehydroascorbate reductase (MDHAR), GSH-dependent dehydroascorbate reductase (DHAR) and glutathione reductase (GR), catalyse the recycling of ascorbate. Superoxide dismutase (SOD) converts O_2_^●−^ into H_2_O_2_. Ascorbate peroxidase (APX) (1), catalase (CAT) (2), and GPX (3) act as the main H_2_O_2_ detoxifying enzymes. AA and GSH are antioxidants. Abbreviations: ascorbate peroxidase (APX), oxidised glutathione (GSH), reduced glutathione (GSSG).

More importantly, APXs deal with H_2_O_2_ signalling during developmental stages [144,145] through scavenging H_2_O_2_ toxicity in the chloroplast and cytosol [26,146]. APX enzymes possess a strong affinity for H_2_O_2_, indicating that the ascorbate-glutathione (AA-GSH) cycle plays a vital role in controlling ROS levels in cellular organelles [138]. The way this enzyme decomposes H_2_O_2_ into water and oxygen is to use ascorbate (ascorbic acid-AA) as a reducing factor [11]. In this reaction, ascorbate is converted to monodehydroascorbate (MDHA) by APX activity, and this compound is also converted to dehydroascorbate (DHA) through a non-enzymatic pathway. Additionally, the ascorbate is a prerequisite substance to maintain the production cycle. In the AA-GSH cycle, monodehydroascorbate reductase (MDHAR) converts the MDHA to AA utilising NADPH. Also, the conversion reaction of DHA to AA is catalysed by dehydroascorbate reductase (DHAR) with redox glutathione (GSH) oxidation in the process (Figure 1) [147].

Similar to the enzyme CAT, APX also presents either in the thylakoid membrane or as a solution in the stroma. The form attached to the thylakoid membrane immediately detoxifies the hydrogen peroxide at the production site, and the stroma-soluble type decomposes the hydrogen peroxide released into the stroma. In comparison to CAT, APX has a stronger affinity for H_2_O_2_; hence it is a better scavenger at times of stress [26]. The noticeable concentration of SOD, CAT and APX in the heavy metal (HM)-resistant plant *Pteris vittata* in proportion to the HM-sensitive plant *Pteris ensiformis* suggests that the so-called enzymes play a central role in the detoxification of zinc oxide nanoparticles [148].

### 2.4. Guaiacol Peroxidase (GPX)

With its 40–50 kDa monomers, this heme-containing enzyme, GPX (EC 1.11.1.7), is capable of eliminating excess H_2_O_2_, whether by normal or stress-driven metabolism [26]. GPX isoenzymes are localised in vacuoles, cell walls and the cytosol (Figure 1) [149]. The two main functions of GPX are lignin biosynthesis and biotic stress defence by virtue of indole acetic acid (IAA) degradation and H_2_O_2_ utilisation in the process [150]. GPX prefers aromatic compounds (e.g., guaiacol and pyrogallol) over non-aromatic ones due to their higher electron-donating ability [151]. As a consequence of its fairly high activity in intra- and extracellular spaces or cell walls, it has been postulated to be a critical enzyme in H_2_O_2_ removal [152]. Because this enzyme undertakes both peroxidative and hydroxylic reactions, it comes as no surprise that it can participate in different plant processes such as incorporation in biosynthetic processes, auxin metabolism, cell wall elongation and lignification, as well as pathogenic resistance [153]. A number of environment-born stress factors including herbicide [154], potentially toxic metals [155,156], polycyclic aromatic hydrocarbon (PAH) [157], and ozone (O_3_) [158] are involved in the induction of GPX activation.

### 2.5. Glutathione Reductase (GR)

This enzyme (GR, EC 1.6.4.2), also known as flavoprotein oxidoreductase, is the last in the ascorbate-glutathione cycle that is found predominantly in chloroplasts (GR_2_) and partially in the cytosol (GR_1_), mitochondria (GR_2_) [11,26,159] and peroxisomes (GR_1_) [11]. The proper enzymatic activity of GR mainly springs from its possession of triple domains, including NADPH-binding domain, FAD-binding domain, and a dimerisation domain [160]. The above-mentioned isozymes of GR are involved in tolerance to high light (GR_1_, GR_2_) [161,162], salt stress (GR_1_) [163,164], chilling stress (GR_2_) [165,166], methyl viologen (MV^2+^)-induced oxidative stress (GR_2_) [167], and toxic metals (GR_1_) [168,169]. Moreover, GR_2_ has been proven to interfere with developmental processes involving embryo development [170], seed germination [171], root growth, and apical meristems maintenance [172]. Thus, it is follows from the above that abiotic [173,174] and possibly biotic [175] stress factors induce the activity of GR in plants.

GR mediated plant tolerance to stress conditions often refers to its role in ROS detoxification through GSH (reduced glutathione) regeneration [176]. GR has been proven to mediate the conversion of oxidised glutathione (glutathione disulfide-GSSG) to GSH, using the electron donor (reductant) NADPH. Moreover, most of the synthesised GSH is involved in the regeneration of ascorbate (AA) from DHA under DHAR mediation (Figure 1) [26], and the activation of several CO_2_-stabilizing chloroplast enzymes [4]. Also, GR has the potential to maintain a high GSH/GSSH cellular ratio by catalysing the formation of glutathione disulfide [26], signifying that the pool of GSH consumed by the DHAR reaction is replenished by GR [11]. Increasing GR activity increases the NADP_+_/NADPH ratio, thus elevating the amount of available NADP_+_ as the last electron receptor in photosynthetic light reactions, and eventually reducing the likelihood of electrons being transferred to O_2_ for superoxide radical production [177,178,179].

### 2.6. Monodehydroascorbate Reductase (MDHAR)

AA regeneration from transient MDHA is catalysed by the FAD (flavin adenine dinucleotide)-dependent MDHAR (E.C.1.6.5.4) enzyme using electrons donated by NADPH [26,180,181]. Notably, MDHAR and APX enzymes are co-localised in the mitochondria and peroxisomes, where their reducing and oxidising activity creates a balance between AA and MDHA pool sizes [10,182]. According to Obara et al. [183], six functional proteins have been encoded from only five *Arabidopsis* genes. MDHAR isozymes are laid out in different subcellular compartments, including mitochondria (MDHAR_5_), peroxisomes (MDHAR_1_) and its membrane glyoxysomes (MDHAR_4_), chloroplast (MDHAR_6_), and cytosol (MDHAR_2_ and MDHAR_3_) [11,184]. Other than their antioxidant potential, not much information is available on the specific function of MDHAR genes. As a case in point, Eastmond [185] suggested the possible interference of MDHAR_4_ in different growth stages of Arabidopsis, from germination to post-germination, through senescence. Based on studies conducted so far on rice, tobacco and Arabidopsis, it was revealed that overexpression of MDHAR genes resulted in an increased tolerance to salt, ozone, and osmotic stress as well as higher germination rate and grain weight [186,187].

### 2.7. Dehydroascorbate Reductase (DHAR)

What is certain in the reduction process of DHA to AA is the DHAR utilisation and transformation of the reduced GSH as an electron donor, which eventually maintains the redox state of plant cells [186,188]. This introduces DHAR as the second catalytic agent along with the so-called MDHAR in regulating the regeneration of the cellular AA pool both in symplast and apoplast [189]. The regulation of AA homeostasis by DHAR during the plant’s developmental processes is no more likely than GSH homeostasis regulation by DHAR [190]. DHAR is usually found in seeds, roots, and shoots with (green) or without (etiolated) chlorophyll content [26]. To date, three types of DHAR proteins have been identified as soluble monomeric enzymes in the chloroplast (DHAR_3_) and cytosol (DHAR_1_, DHAR_2_) [11,188]. A plethora of studies have defined various functions for DHARs at their site of localisation, where, for example, DHAR_1_ is involved in plant responses to high light, high temperature, and MV^2+^-induced oxidative stresses [167,191,192], DHAR_2_ in plant protection against Polyethylene glycol (PEG), salt, drought, and ozone [186,193], or DHAR_3_ in high light response conference [194].

## 3. Non-Enzymatic Defensive Mechanisms

Non-enzymatic antioxidants are low molecular weight molecules with specific structures, chemical properties, and locations. Possessing a pivotal role in eliminating free radicals by virtue of donating electrons or hydrogen, these compounds are divided into two groups [4,26]: (1) Fat-soluble membrane antioxidants such as α-tocopherol, carotenoids and xanthophyll; (2) Water-soluble antioxidants such as glutathione, ascorbate and phenolic compounds [195].

### 3.1. Ascorbic Acid

As one of the most abundant antioxidants in plant systems, ascorbic acid ((AA),vitamin C) has a critical function in plant growth and development [196]. The metabolisation of ROS species by the redox buffer AA could have been explained by safeguarding cells against free oxygen radicals generated under different environmental stress factors [197]. Usually, continuous oxidation of AA is observed under the disguise of such scavenging [196]. Different pathways are in place to ensure the recycling of AA in spite of its slow and time-consuming biosynthesis [196]. Two key enzymes are involved in maintaining the AA homeostasis in living organisms under solicited and unsolicited exogenous stimuli; MDHAR and DHAR [198]. The oxidised (MDHA, DHA), or reduced (MDHAR, DHAR) forms of AA are tapped in AA regeneration [199]. As primary products of AA oxidation, the MDHA molecules can bind each other to form AA or DHA [196]. There has always been the possibility of generating an irreversible form of 2,3-diketogulonic acid in the course of spontaneous DHA hydrolysis [198]. Notably, NAD(P)H figures as an MDHAR electron donor, reducing MDHA to AA in an enzymatic reaction [200].

DHA reduction to AA is mediated by the coupled activity of DHAR and GSH (hydrogen donor) oxidation (Figure 1). There are positive reports on the impact of DHAR overexpression on improved grain and biomass yield in several plants [193,201,202,203,204]. For instance, Kim et al. [204] reported that transgenic *japonica* rice genotypes with high DHAR expression had increased concentrations of AA and higher crop productivity than wild-type rice. Based on these results, one may speculate on the existence of a direct relationship between AA pools and the grain yield and decreased ROS in plants lacking sufficient environmental adaptation. DHAR down-regulating tobacco plants have shown lower ascorbate levels, signifying the importance of DHAR proteins in AA regeneration [205]. An opposite trend in DHAR expression, however, led to higher AA (oxidised) and GSH (reduced) concentrations [198]. Despite the above statements, a recent finding by Rahantaniaina et al. [188] demonstrated that there is not much difference between an Arabidopsis (*Arabidopsis thaliana*) mutant without all three DHAR proteins and a wild-type plant with respect to AA regeneration levels, as well as of growth and development. In addition to the analysis of DHAR mutants that show DHARs are required to tie the H_2_O_2_ metabolism with GSH oxidation, this finding has brought uncertainty in the efficiency of DAHRs in AA recycling [197].

Physiological studies demonstrate that DAHRs function properly in the course of ascorbate oxidation and increase in ascorbate pool size in a state of high-light stress conditions [206,207]. A study on the Arabidopsis *Ddhar* mutant identified that the DHAR proteins contributed to increased growth and ascorbate homeostasis under low-light stress [197]. Under high-light stress, both the *Ddhar* mutant and wild-type plants elevate their ascorbate concentration, with a minor reduction seen in the ascorbate accumulation of *Ddhar* compared to the wild type that was ascribed to an ascorbate degradation product, the threonate [207]. Accordingly, the authors assumed that the ascorbate pool size determined the activity range of DAHRs and that the necessity of DAHRs activity (to regenerate ascorbate) was a function of higher ascorbate levels. In other words, the smaller the Arabidopsis pool of ascorbate, the lower the DAHRs regeneration activity to sustain ascorbate recycling. It appears that the non-enzymatic reduction of AA by GSH can act as a backup to maintain DAHR activity [197]. In agreement with earlier results [208], a recent report shows that only 30% of the average GSH level in a wild type plant is sufficient to sustain AA recycling under high-light stress conditions [197]. However, an appreciable reduction in AA accumulation as a result of prolonged high-light stress and/or pharmacologically induced GSH deficiency was noticed in the *Ddhar* pad2-1 quadruple mutants. This was attributed to lower photochemical activity, bleaching, and increased accumulation of threonate [197]. This study put forward the hypothesis that glutathione compensates for the loss of DAHR function under high-light conditions [207]. In the case of Arabidopsis, the activity of DAHR proteins is necessary on the condition that the accumulation level of AA is high (high light) or GSH is less available [207]. Unlike the earlier reports on the indispensable role of DAHRs on ascorbate recycling in tobacco [189,205], recent studies in Arabidopsis underscore the neutral function of DAHRs in ascorbate recycling or maintaining its redox state without affecting ascorbate levels [188].

Even though AA is naturally synthesised in the inner membrane of mitochondria, it may also be found in the cytosol, cell walls, chloroplasts, vacuoles, and apoplasts [209]. AA performs different roles in cells, such as controlling cell cycles, growth and development, and not least having effects on cell wall elongation and the redox level adjustment [196,199]. Ascorbate is a precursor to oxalate and tartrate as well as a cofactor of enzymes involved in the synthesis of glycoproteins rich in hydroxyproline (Hyp), ethylene, gibberellin, and anthocyanin [196]. In addition to being known as a substrate of many peroxidases, AA has been shown to be one of the main components of the ascorbate-glutathione and/or water–water cycle that effectively eliminates ROS [4]. Also, the possibility of AA oxidation in direct reaction with active oxygen species, such as superoxide, singlet oxygen, or hydroxyl radicals, or AA utilisation as a reducing agent in α-tocopherol regeneration to protect membranes from oxidative stress was reported earlier by Parida and Das [147]. Another point is that AA in conjunction with α-tocopherol scavenges lipid peroxyl radicals and prevents the spread of lipid peroxidation in membranes [210,211]. As an example, in chloroplasts, AA functions as a cofactor for the enzyme violaxanthin de-epoxidase (VDE) and participates in the distribution of excessive excitation energy [4].

### 3.2. Glutathione

Glutathione (GSH) is an intact *tripeptide*, *α-glutamylcysteinyl glycine* present in all cell parts, including cytosol, chloroplast, endoplasmic reticulum vacuole and mitochondria, with the highest amount reported in the chloroplast [26,212]. The GSH pool is an essential component of the cellular redox system, which effectively controls the amount of H_2_O_2_ through the ascorbate–glutathione cycle and the glutathione cycle [4]. Furthermore, the regeneration process of ascorbate during the ascorbate–glutathione cycle is exclusively premiered on the functioning of the GSH [147]. The antioxidant GSH can act as an eliminator of ROS species. In addition to interfering with the antioxidant defence system, glutathione may also be involved in regulating other processes such as cell entry into the G_1_ phase and cell differentiation and death. It is believed that this compound is one of the primary sources of non-protein thiols in most plant cells. Given the high reactivity domain of thiol (Sulfhydryl -SH) groups of the low molecular weight compound, GSH, these substances are tapped into many chemical reactions [26]. The nucleophilic nature of the thiol group is critical in the formation of bonds with metals and electrophilic materials. For instance, GSH has been found to positively impact phytochelatin formation via phytochelatin synthase [213]. This reactivity, combined with high stability and solubility of glutathione in water, makes it an ideal substance for protecting plants against environmental stimuli such as heavy metal stress [26,214]. Another beneficial aspect of this molecule is the high reduction potential of the glutathione molecule thanks to its central (C-terminal) cysteine residues [4].

### 3.3. Proline

The amino acid proline has also been regarded as a non-enzymatic antioxidant in plant systems that can easily counteract the harmful effect of ROS [26,215,216]. Proline synthesis from substrate glutamate (glutamic acid) is a consecutive reaction catalysed by two enzymes, ð1-pyrroline 5-carboxylate (P5C) synthase (P5C-S) and P5C reductase (P5C-R) [215]. The involvement of proline in scavenging ROS damage through direct reaction with ROS has been widely investigated [217,218,219]. For example, some studies revealed that proline osmolytes containing polypeptides could react with H_2_O_2_ and OH^•^ to generate stable free radicals by adducting to prolines and hydroxyproline derivatives such as as 4-hydroxyproline and 3-hydroxyproline [217,220]. Further observations by Kaul et al. [218] indicated that proline indirectly scavenges the cellular H_2_O_2_ or O2^•−^. In *Brassica juncea* plants, proline involvement could dramatically suppress the production of ^1^O_2_ in the thylakoids [221]. The ^1^O_2_ quenching feature of prolines has been suggested to help stabilise proteins, DNA, and membranes [222]. One of the attractive functions of prolines is to restore the cellular redox balance disrupted by ROS during heavy metal stress [219]. Sharma et al. [223] reported that proline has the potential to prevent zinc (Zn) and cadmium (Cd) from reducing the activity of the cellular enzymes by forming complexes with these metals. Similar protection was earlier observed in copper (Cu)-tolerant *Armeria maritime* (Mill.) Willd [224]. Multiple studies have identified the progressive impact of proline on antioxidant activities of defensive enzymes such as CAT-in oxidative stress [225,226], SOD- in Cd stress [227], or enzymes related to GSH- in salt stress [228], and AA-GSH cycle- in Cd stress [229].

### 3.4. α-Tocopherols

*α-*tocopherol with its three methyl substituents is considered to be one of the most reactive antioxidants among the four well-known lipoperophilic isomers of tocopherol (α, β, γ, and δ) [26]. Regardless of present tocopherol groups, they are responsible for scavenging lipid peroxy radicals, oxygen free radicals and singlet oxygen [230,231,232]. Tocopherols are often localised in the green tissues of plants where photosynthetic organelles and pigments are found [152]. After tocopherol synthesis is completed in the inner envelope of chloroplasts, it has been proposed that they transfer and accumulate in all chloroplast membranes [233,234]. Given that tocopherols are generally incorporated in the protection of lipids, and proteins and pigments are incorporated in the photosynthetic apparatus against oxidative stress, loss of them would be expected to adversely affect growth and photosynthesis in plant systems [235]. There are indications showing that tocopherols’ presence in chloroplasts reinforced plant tolerance to salinity, chilling and water deficit stresses [236,237,238,239].

A combination of five catalysing enzymes (i.e., 4-hydroxyphenylpyruvate dioxygenase, 2-methyl-6-phytylbenzoquinol methyl-transferase, homogentisate phytyl transferases, γ-tocopherol methyl-transferase, tocopherol cyclase (VTE_1_), and two precursors (homogentisic acid and phytyl diphosphate) compounds are required for tocopherol biosynthesis [240]. Notably, the enzyme γ-tocopherol methyl-transferase (γ-TMT) has been found to catalyse the biosynthesis of *α-*tocopherol from γ-tocopherol [26,241]. The penultimate step in tocopherol biosynthesis in leaves is dependent on the availability of the VTE1 enzyme [235]. This suggests that VTE_1_ deficiency would result in a significant reduction in tocopherol synthesis [242].

The way tocopherols are utilised to indirectly protect the structure and function of photosystem II (PSII) is to establish a chemical reaction with O_2_ and physically quench its excess energy, therefore protecting lipids and other components in the double-membrane of chloroplasts [243]. The prevention of membrane lipid autoxidation through *α-*tocopherols interaction with lipid radicals (i.e., RO^•^, ROO^•^, and RO^*^) has identified them as protectors of biological membranes [241,244]. On the other hand, the free radical trapper *α-*tocopherol represses the chain propagation step in the lipid peroxidation (LPO) cycle [26,152,245]. Cellular regeneration of oxidised tocopherols (TOH^•^) is often driven by coenzyme Q [246] or AA and GSH [247,248]. More specifically, the benzoquinone ring (after full substitution) or the phytyl chain (after full reduction) in tocopherols may act as an efficient antioxidant for ^1^O_2_ neutralisation [249,250,251]. In addition to the inhibition of non-enzyme based LPO under stress conditions [241], *α-*tocopherols are capable of protecting seed storage lipids, activating plant defence responses, functioning in membrane stability, or involving in seedling germination, transcript regulation and intracellular signalling [252,253,254].

### 3.5. Carotenoids

Carotenoids are tetraterpene antioxidants found in the plastids of photosynthetic and non-photosynthetic tissues of plants [255,256] and are synthesised by geranyl pyrophosphate (GPP) in the course of isoprenoids [26]. Carotenoids containing pure hydrocarbons (carotene) and those with one or more oxygen atoms (xanthophyll) are two main types of carotenoids in plant tissues [257]. Apart from their presence in plants, carotenoids have also been found in algae and photosynthetic microorganisms [152,256]. Their light-harvesting behaviour in chloroplasts encompasses not only light absorption by antenna molecules (450–570 nm) and transfer of energy to chlorophyll (Chl) molecules but also photosynthetic machinery protection [258,259,260]. Carotenoids exert their antioxidative functioning in photosynthetic apparatus through scavenging singlet oxygen activity, reacting with LPOs to halt the chain reaction of ROS production, quenching triplet (3Chl*), and exciting (Chl*) Chl molecules to prevent the formation of singlet oxygen, and dissipating excess excitation energy in the xanthophyll cycle [26,261,262,263]. Depending on plants’ resistance threshold to stressful conditions, the amounts of carotenoids are affected [264,265]. Sugarcane plants with high carotenoids contents exhibited a better adaptation to saline conditions [266].

### 3.6. Phenolic Compounds

Flavonoids, anthocyanins, tannins, hydroxycinnamic acid esters, and lignins are phenolic compounds that belong to the secondary metabolites arising from the phenylpropanoid pathway in plant tissues [267]. The enzyme phenylalanine ammonia-lyase (PAL) is the tropical initiator of phenylpropanoid, which converts L-phenylalanine to trans-cinnamic acid by deamination. This pathway is necessary for the biosynthesis of secondary metabolites in living cells [268]. Phenolic compounds are naturally synthesised in the cell under optimal conditions, but when there is biotic or abiotic stress, the concentration of these products are significantly affected [152]. Also, any alteration in the activity of biosynthesising or degrading enzymes may influence the amount of these compounds in plant cells.

Flavonoids are a large group of secondary metabolites that are widely distributed among plants and have multiple roles, including contributing to colouring of flowers, seeds and pollen grains, helping in pollination, germination and pollen tube growth, and auxin transport [26], as well as inducing protection against photosynthetic damages caused by excess excitation energy [269]. The latter function is presumably associated with its ROS scavenging capacity [152]. Thanks to the presence of flavonoids, living cells are capable of alleviating the damage of ^1^O_2_ on the outer envelope of the chloroplastic membrane [269,270]. The main enzymes involved in the biosynthesis of flavonoids are PAL and chalcone synthase (CHS). Until now, about 12 groups of flavonoids have been identified, the three most important of which are flavonoids, flavonols and anthocyanins [271]. Aside from the roles mentioned above, phenolic compounds also have an antioxidant property in the cell.

Considering the fundamental role of phenolic compounds in reducing or inhibiting lipid auto-oxidation, eliminating oxygen free radicals, quenching singlet oxygen or decomposing peroxides, they have also been known as essential antioxidants responsible for protection against proliferation and advancement of the oxidation chain and defence against reactive oxygen species [271]. The antioxidant properties of phenolic compounds are tied to their chemical structure that may act as an electron or H^+^ donor. Polyphenols have been shown to chelate intermediate metals such as iron, thus preventing the Fenton and/or Haber–Weiss reaction [272]. Numerous studies in plants demonstrated the impact of potentially toxic heavy metals on the amounts of phenolic compounds, glutathione, phytochelatins, ascorbate, carotenoids, anthocyanins and the activity of PAL enzyme. For instance, the accumulation of these compounds in the presence of zinc oxide nanoparticles was reported [273].

## 4. Antioxidant Machinery in Plant Systems and Microbial Mediation in Promoting Plant Tolerance

ROS signals are initially perceived and transduced in plants before being translated into sufficient responses [11]. The oxidising nature of ROS aggregates determines the modification/modulation level of potential signalling targets such as transcription factors, kinases, and stress-induced proteins. Notably, this modulation is subject to ROS capability in affecting the protein redox status through oxidation of thiol groups and methionine residues [274]. Thio- and gluta-redoxins are two examples of proteins with the capacity to regulate cellular redox states via their interactive activation/deactivation or reversible oxidation/reduction [275]. For instance, a redox-sensing mechanism was introduced for apoplastic H_2_O_2_ perception and transduction by Wu et al. [276]. Protein oxidation or the attachment of carbonyl groups (ketones and aldehydes) to the protein side chains of threonine, lysine, proline, or arginine is known as ROS-mediated carbonylation [277]. This process might lead to protein instability and susceptibility to proteolysis [278,279]. ROS-driven redox perturbations have been found to be transduced by metabolic signals to switch on rapid adaptive mechanisms by mitochondrial/chloroplastic retrograde signalling [280]. Also, ROS can mediate the plastids to the nucleus retrograde-signalling pathway [11]. As such, the nucleus can host the H_2_O_2_ generated in plastids at the expense of activating the defence gene expression [281]. There are few reports showing that ROS can interplay with other secondary messengers, such as reactive nitrogen species (RNS) and Ca^2+^ [11,282,283]. Thanks to their high oxidative potential, ROS interact with nitric oxide (NO) messengers, leading to the generation of (non-)radical RNS products including nitrous acid (HNO_2_), nitroxyl anion (NO^−^), nitrosonium cation (NO^+^), peroxynitrite (ONOO^−^), nitrate (NO^•^_3_), nitric oxide (NO^•^), and nitric dioxide (NO^•^_2_) [284,285,286,287]. These NOx species by nature are involved in plant development, metabolic processes, stress-dependent responses and stomatal closure [288]. Delledonne et al. [289] were the first to report the existence of an interplay between NO and H_2_O_2_ during plant hypertension responses. Usually, the crosstalk of ROS and RNS accompanies, by direct or indirect modulation, antioxidant enzymes [285] and may have deleterious or beneficial effects on plant cells, and is highly dependent on the concentration and specific subcellular microcompartment/organelle type [286,290,291].

Increased ROS generation as a result of different environmental stresses has always been naturally responded to in biological systems in multiple ways under the disguise of antioxidative defence mechanisms [11,292]. In addition to this, mitigating the damaging consequences of adverse environmental conditions via the generation of ROS-response antioxidants can also occur by exogenous factors such as plant growth-promoting rhizobacteria (PGPR) [293,294], and plant growth-promoting fungi (PGPF) [295]. It has been suggested that growth-promoting bacteria have the potential to confer enhanced tolerance to abiotic stresses through the induction of physical and chemical alterations in planta, a mechanism that offers protection and is referred to as PGPR-induced systemic tolerance (IST) [296,297,298,299,300,301,302,303]. However, in the case of biotic stress, the so-called eliciting function of PGPRs is known as induced systemic resistance (ISR) [304,305].

The PGPRs function in inducing plant tolerance against abiotic stresses, such as salt, drought, and extreme temperatures, is of key importance in alleviating the adverse effect of climate change on sustainable crop production [299]. As outlined earlier in this review, Wang et al. [45] have shown that the inoculation of *Cucumis sativus* (cucumber) with a consortium of three PGPR strains could induce systemic tolerance in drought-imposed plants by maintaining root vigour, photosynthetic performance, and increased generation and activity of SOD, CAT and prolines in the leaves. In *Lycopersicon esculentum* (tomato) plants treated by a *Bacillus cereus* AR156 supernatant, an appreciable enhancement to drought stress was reported by Wang et al. [306]. The induced tolerance was associated with the increased chlorophyll a and b contents, as well as the enhanced activities of CAT, SOD, and peroxidase (POX). Enhanced salinity tolerance in *Panicum turgidum* Forssk plants has been attributed to the *Arbuscular mycorrhizal* fungi contribution by modifying photosynthetic and antioxidant pathways [307]. Similarly, PGPR alleviated drought stress in potato (*Solanum tuberosum* L.) plants treated with *Bacillus subtilis* HAS31 by maintaining higher photosynthetic processes, total soluble sugars, proteins and prolines with elevated activity of POX, CAT, and SOD [281]. In another study by Banik et al. [308], it was revealed that PGPR treatment significantly augmented drought tolerance in *Agrostis palustris* by lowering MDA accumulation and developing osmotic adjustments associated with higher synthesis and accumulation of compatible solutes, including soluble sugars, free amino acids, proteins, and not least the non-enzymatic prolines. Plant inoculation with PGPRs adds up to the available quantity of proline in plant systems under stress conditions [309]. As such, a sizable quantity of prolines was increased in *Zea mays* L. after inoculation with *P. fluorescens* under drought stress [310]. A noticeable enhancement in drought tolerance of *Lavandula dentate* plants treated with PGPR *B. thuringiensis* was recorded under drought conditions, which was attributed to the increased shoot proline contents [311]. Also, there was an excess secretion of proline in the root of tomato (*Lycopersicon esculentum* Mill cv. Anakha) plants after exposition to *Bacillus polymyxa* [312]. Similar to plants, PGPRs secrete osmolytes (e.g., proline) at the time of water scarcity, which cumulatively helps in stimulating plant growth [313].

The application of *Trichoderma afroharzianum* strain T22 has been shown to enhance tomato (*Solanum lycopersicum* L.) seed germination under biotic stress conditions by alleviating oxidative damage in salt-stressed seedlings [314]. Similarly, Zhang et al. [282] demonstrated that the PGPF *Trichoderma longibrachiatum* strain T6 induced the tolerance of wheat seedlings to salt stress through upregulation of SOD, CAT and POX genes, improving the antioxidant defence machinery. Compared to uninoculated rice genotypes Swarna and Swarna sub-1 under severe drought conditions, the PGPFs (*Fusarium pallidoroseum* strain-10 and *Trichoderma harzianum* strain-35) inoculated rice genotypes showed higher activity of SOD, POX, and CAT [315]. Table 2 briefly presents the antioxidative responses in selected plant species after exposure to various environmental stresses.

## 5. Conclusions and Future Prospects

ROS are natural byproducts of many metabolic pathways or their respective electron transport activities in different cellular compartments. Thanks to cellular auto adjustment mechanisms, there is always a homeostatic balance between ROS production and removal machinery which make ROS less harmful to plants in normal environments. However, prolonged environmental stresses such as salinity, chilling, drought, water deficit, and UV radiation can severely exacerbate the production level of ROS by disrupting the natural cellular homeostasis, changing the ROS role as a signalling molecule to a damaging oxidant capable of harming lipids, proteins and DNA. Plants have developed different enzymatic and non-enzymatic antioxidative pathways to alleviate the adverse effects of oxidative damages. The term Induced Systemic Tolerance (IST) has been coined in this review to capture the concept of microbial modulation of enhanced plant tolerance against abiotic stresses through inducing physical and chemical alterations. The beneficial microbiome related to roots and plant tissues suppresses plant stress by a variety of processes. As an example among them, PGPRs are capable of enhancing plant micronutrient uptake, regulating phytohormones homeostasis, and stimulating the plant immune system against biotic and abiotic stresses.

Insofar as the results of ROS experiments under a variety of imposed stresses are concerned, it is not entirely clear how plants deal with stress-induced ROS homeostasis disruption and the consequent cellular degradation. There are still many unsolved questions regarding the simultaneous antioxidative activities of enzymatic antioxidants and their non-enzymatic counterparts. These uncertainties can be considered as hints for further studies in ROS formation and ROS removal machinery. In light of ROS studies, the use of state of art analytical and/or imaging techniques might provide a broader insight into understanding the complex antioxidant networks involved in plant responses to elevated ROS levels. Further, the combined application of advanced functional genomics, proteomics and metabolomics might be helpful in ROS network elaboration. Transgenic techniques can also be used for producing plants with high tolerance to multiple stresses.

## Figures and Tables

**Table 1:** CAT cDNA isolated and characterised from different plant species.

Plant Species	Common Name	Genomic DNA or Protein	Subcellular Localisation and Site of Detection in Plant	References
*Brassica napus*	Rapeseed	CAT_1_–CAT_14_	Peroxisomes (CAT_2_–CAT_3_, CAT_6_–CAT_8_, CAT_11_–CAT_14_), cytoskeleton (CAT_1_), cytoplasm (CAT_4_), mitochondrion (CAT_9_), and chloroplast (CAT_5_, CAT_10_) in root, leaf, stem, and silique samples	[89]
*Helianthus annus*	Sunflower	CAT_1_–CAT_8_	Peroxisomes in cotyledons and roots	[90,91,92]
*Gossypium hirsutum*	Cotton	CAT_1_–CAT_7_	Peroxisomes in leaves	[78,93]
*Pisum sativum*	Pea	CAT_1_–CAT_5_	Peroxisomes in leaves and the whole fruit	[94,95]
*Cucumis sativus*	Cucumber	CAT_1_–CAT_4_	Peroxisomes in roots, stem, leaves, flowers, and fruits	[79]
*Oryza sativa* L.	Rice	OsCATA–OsCATD	Cytosol (OsCATA–OsCATC), peroxisomes (OsCATB, OsCATC), and plasma membrane (OsCATD) in immature seeds and rice seedlings	[80,96,97]
*Nicotiana plumbaginofolia*	Tex-Mex tobacco	CAT_1_–CAT_3_	Peroxisomes in leaves	[84]
*Arabidopsis thaliana*	Arabidopsis	CAT_1_–CAT_3_	Peroxisomes in bolts, leaves, siliques (CAT_1_, CAT_2_), in pollen and seeds (CAT_1_), stems and roots (CAT_3_)	[81,98,99,100,101]
*Cucurpita pepo*	Pumpkin	CAT_1_–CAT_3_	Glyoxysomes in seeds and early seedlings (CAT_1_), mature leaves, stem (CAT_2_), green cotyledons and green hypocotyls (CAT_2_, CAT_3_), and roots (CAT_3_)	[82]
*Glycine max*	Soybean	CAT_1_–CAT_3_	Glyoxysomes in developing kernels (CAT_1_), green leaves (CAT_2_, CAT_3_), roots (CAT_3_), and epicotyl of the developing seedlings (CAT_3_)	[102]
*Zea mays*	Maize	CAT_1_–CAT_3_	In scutella, milky endosperm of immature kernels, leaves and epicotyls (CAT_1_); Post-germinative scutella, with lower levels in leaves, epicotyls and growing kernels (CAT_2_); Epicotyls and, to a lesser extent, in leaves and scutella (CAT_3_)	[83,103,104,105,106,107]
*Capsicum annuum*	Hot pepper	CAT_1_–CAT_3_	Peroxisomes in leaf and stem	[108]
*Nicotiana tabacum*	Tobacco	CAT_1_–CAT_3_	Peroxisomes in leaves	[109,110]
*Ricinus cammunis*	Castor bean	CAT_1_, CAT_2_	Glyoxysomes, peroxisomes in hypocotyls and roots (CAT_2_), in endosperms and cotyledons (CAT_1_)	[88,111]
*Hurdeom vulgare*	Barley	CAT_1_, CAT_2_	Peroxisomes in whole endosperms, in isolated aleurones and in developing seeds (CAT_1_), in etiolated seedling shoots and leaf blades (CAT_2_)	[85]
*Triticum aestivum*	Wheat	CAT_1_, CAT_2_	Peroxisomes in leaves	[112]
*Solanum tuberosum*	Potato	CAT_1_, CAT_2_	Peroxisomes in leaves	[113,114]
*Lycopersicum sculentum*	Tomato	CAT_1_, CAT_2_	Peroxisomes in leaves	[86,115,116]
*Nicotiana sylvestris*	Woodland tobacco	CAT_1_, CAT_4_	Peroxisomes in leaves	[117]
*Vigna radiata*	Mung bean	CAT_1_	Glyoxysomes, leaf peroxisomes, and nonspecialised microbodies (etiolated or nongreen tissues)	[118]
*Secale cereale*	Rye	CAT_1_	Peroxisomes in leaves	[119]
*Ipomoea batatas*	Sweet potato	CAT_1_	Peroxisomes in leaves	[87,120]

**Table 2:** Enzymatic/non-enzymatic antioxidants reported in selected plant species under stress conditions.

Plant Species	Stress Type	Antioxidant Reported + (Increase)/−(Decrease)	Reference
*Phaseolus vulgaris* L.	Drought	+SOD, +APX in leaves	[316]
*Trifolium repens* L.	Drought	+SOD in leaves	[317]
*Cicer arietinum* L. cv. ILC482	Drought	+Proline in leaves	[318]
*Cicer arietinum* L. cv. *CSG-8962*	Salinity	+SOD, +CAT, + Peroxidase (POX), +APX, +GR, and –AA in roots	[319]
*Cicer arietinum* L. cv. Gökçe	Salinity	+SOD in roots and leaves, +APX and +GR in leaves, and +CAT in roots, –CAT in leaves	[320]
*Lycopersicon esculentum* Mill. cv. Perkoz	Salinity	+SOD, +Glutathione S-transferase (GST), and +Glutathione peroxidase (GSH-PX) in roots	[321]
*Withania somnifera* L.	UV-B radiation	+CAT, +GR, +POX, +Polyphenol oxidase (PPO), and +SOD in roots > leaves	[322]
*Vicia sativa* L.	Cd stress	+SOD, +APX, and +CAT in roots	[323]
*Pisum sativum* L.	Cd stress	–SOD, –CAT, –GPX, and +Lipid peroxidation (LPO) in leaves	[324]
*Hibiscus cannabinus* L. cvs. Fuhong 991 and ZM 412	Cd stress	+GR in leaves (Fuhong 991), +SOD, +CAT, and +POX in roots (Fuhong 991 and ZM 412), +Lipid peroxidation (LPO) in roots (ZM 412)	[325]
*Jatropha curcas* L.	Salinity	+SOD, +CAT, +POX in the cotyledons, hypocotyls and radicles	[326]
*Nicotiana tabacum* L. line Chl-APX5	Salinity and drought	+APX in chloroplast	[327]
*Nicotiana tabacum* L.	Salinity	+SOD, +CAT, +APX, +POX, and +GR in roots	[328]
*Nicotiana tabacum* L.	Salt, O_3_ and polyethylene glycol (PEG) stresses	+MDHAR in leaves	[186]
*Arabidopsis thaliana* L.	Salinity	+DHAR in leaves	[201]
*Arabidopsis thaliana* L.	Salinity	+SOD, +CAT, and +POX in leaves	[329]
*Arabidopsis thaliana* L.	UV-B/Ozone (O_3_) radiation	+SOD, +APX, +GPX, +POX (UV-B and O_3_), and +GR (O_3_) in leaves	[330]
*Eichhornia crassipes* (water hyacinth)	Pb stress	+APX, +POX, +SOD, +CAT in leaf and root tissues	[331]
*Vigna radiata* L. Wilczek	CdCl_2_ stress	+MDHA +POX, +CAT, and +Proline in roots and leaves	[332]
*Vaccinium myrtillus* L.	Heavy metal stress (Cd, Pb, and Zn)	+GSH, +Non-protein thiols, +Proline, and +GPX in leaves	[333]
*Solanum tuberosum* L.	Salt stress	+APX, +CAT and +GR in roots and shoots	[334]
*Oryza sativa* L.	Drought	+SOD, +APX, +MDHAR, +DHAR, +GR, and –CAT in roots and leaves	[335]
*Oryza sativa* L. cvs. IR-29 and Pusa Basmati	Drought	+Flavonoids, +Phenolics, –CAT, –SOD, and +GPX in leaves	[336]
*Oryza sativa* L. vars. IR-29 and Nonabokra	CdCl_2_/NaCl stress	+ Anthocyanin, + Carotenoids, +GPX, +APX, +Proline, +Polyamines (spermidine and spermine) (IR-29/Nonabokra), –Cysteine and –CAT (IR-29), +Cysteine and +CAT (Nonabokra) in leaves	[337,338]
*Oryza sativa* L. cv. Pokkali	Salt stress	+CAT, +AA, and +GSH in leaves	[339]
*Oryza sativa* L. cvs. CSR-27 and Osmancık-97	Salt stress	+SOD, +APX, +GPX, +CAT, +MDHAR, +DHAR, +GR, and +Proline in roots and leaves	[340,341]
*Oryza sativa* L. vars. Jasmine (KDML105) and Sangyod (SY)	Salt stress	+Flavonoids, and +Proline in shoots	[342]
Turfgrass species	Heat stress	+GSH, and +AA in leaves	[343]
*Lens culinaris* Medik.	Heat stress	+CAT, +APX, +SOD, and +LPO in leaves	[344]
*Triticum aestivum* L. cv. C306	Drought	+SOD, +APX, +CAT, and +AA in leaves	[345]
*Triticum aestivum* L. cv. Chakwal-50	Drought	+Proline,+CAT, +SOD, +POX, and +MDHAR in leaves	[346]
*Triticum aestivum* L. cv. Hindi62	Heat stress	+GSH, +SOD, +CAT, +APX, +GR, and +MDHAR in leaves	[347]
*Triticum aestivum* L.cv. C 306.	Heat stress	+SOD, +APX, +CAT, +GR and +POX in leaves	[348]
*Brassica juncea* L.	High temperature and salt stress	+SOD, +CAT, +APX, +GR, +DHAR, and +MDHAR in seedlings	[349]
*Zea mays* L.	Heat stress	+CAT, +APX, +GR, +AA, +GSH, and +Proline in leaves	[350]
*Lavandula angustifolia cv. Hidcote, Lavandula angustifolia cv. Munstead, and Lavandula stricta*	Drought	+Proline, +CAT, +APX, +POX, +SOD, +MDHA, +Flavonoids, and +Phenols in leaves	[351]
*Rosmarinus officinalis* L.	Drought	+Carotenoids, and +α-tocopherol in leaves	[352]
*Cucumis sativus* L.	Heat stress	+SOD, +CAT, and +POX in leaves	[353]
*Glycine max* L. cv. Clark	UV-B radiation	–APX, –CAT, +SOD, +DHA, and +Flavonoid in leaves	[354]

## Data Availability

No associated data marked.
